# CLPM: A Cross-Linked Peptide Mapping Algorithm for Mass Spectrometric Analysis

**DOI:** 10.1186/1471-2105-6-S2-S9

**Published:** 2005-07-15

**Authors:** Yong Tang, Yingfeng Chen, Cheryl F Lichti, Roger A Hall, Kevin D Raney, Steven F Jennings

**Affiliations:** 1 Department of Applied Science, University of Arkansas at Little Rock, Little Rock, Arkansas 72204; 2 MidSouth Bioinformatics Center, University of Arkansas at Little Rock, Little Rock, Arkansas 72204; 3 Department of Biochemistry and Molecular Biology, University of Arkansas for Medical Sciences, Little Rock, Arkansas 72205

## Abstract

**Background:**

Protein-protein, protein-DNA and protein-RNA interactions are of central importance in biological systems. Quadrapole Time-of-flight (Q-TOF) mass spectrometry is a sensitive, promising tool for studying these interactions. Combining this technique with chemical crosslinking, it is possible to identify the sites of interactions within these complexes. Due to the complexities of the mass spectrometric data of crosslinked proteins, new software is required to analyze the resulting products of these studies.

**Result:**

We designed a Cross-Linked Peptide Mapping (CLPM) algorithm which takes advantage of all of the information available in the experiment including the amino acid sequence from each protein, the identity of the crosslinker, the identity of the digesting enzyme, the level of missed cleavage, and possible chemical modifications. The algorithm does *in silico *digestion and crosslinking, calculates all possible mass values and matches the theoretical data to the actual experimental data provided by the mass spectrometry analysis to identify the crosslinked peptides.

**Conclusion:**

Identifying peptides by their masses can be an efficient starting point for direct sequence confirmation. The CLPM algorithm provides a powerful tool in identifying these potential interaction sites in combination with chemical crosslinking and mass spectrometry. Through this cost-effective approach, subsequent efforts can quickly focus attention on investigating these specific interaction sites.

## Background

Proteins function through complex interactions with other proteins, DNA and RNA and these interactions play a central role in all biological processes. The study of these interactions is an essential requirement to understanding these processes [1-4]. Since errors in these interactions can manifest in disease, potential targets for therapeutic intervention may be identified through these efforts [2,5-7].

A number of approaches have been developed to analyze these associations. These include older, time-consuming, relatively low-throughput methods such as "two-hybrid" and more established methods such as X-ray crystallography and Nuclear Magnetic Resonance (NMR) spectroscopy. These tactics have provided insight into the spatial and topological organization of proteins and protein complexes. However, NMR spectroscopy needs large quantities of purified protein in specific solvents, whereas X-ray studies require that the protein be crystallizable [4].

A more recent strategy based on immuno-affinity, purifies the non-covalently-interacting complex and sequentially analyzes it using mass spectrometry. While this has been proven to be a powerful method for identifying the complex of proteins [8-12], it doesn't provide specific information about the interacting surfaces. However, because of its ultra-sensitive and high-throughput features, mass spectrometry has become the method of choice in the study of proteins and protein complexes [1,2]. Among the several kinds of mass spectrometry, ElectroSpray Ionization (ESI) and Matrix-Assisted Laser Desorption Ionization (MALDI) are the most widely used [13-17].

In order to identify the interacting sites of these complexes, several labs have reported that chemical crosslinking helps to covalently stabilize them. These complexes may then be subjected to gel electrophoresis (to separate crosslinked species) and enzymatic digestion (to yield crosslinked and uncrosslinked peptides). The resulting peptides are then analyzed by mass spectrometry [3,4,16-21]. Chemical crosslinking is also used to study a protein's three-dimensional structure by forming intra-molecular crosslinks [3,22-24]. Unfortunately, the complexities of the mass spectrum of the crosslinked species have been exacerbated by the introduction of the crosslinker.

Some currently-available software such as MASCOT, which identifies proteins from primary sequence databases [25], and SALSA, which detects electrophile-adducted peptides by pattern recognition [26], are oriented towards analyzing uncrosslinked data. Sandia National Laboratories provides a free, web-accessible software package, Automated Spectrum Assignment Program (ASAP) [27] for analyzing chemically-crosslinked protein data from mass spectrometry. With ASAP, however, it is impossible to calculate the cross-linking products from peptides derived from more than one protein and the maximum number of modifications is limited to four [3,27]. For the analysis of protein-oligonucleotide interactions, initiating the crosslinked complex by photochemical crosslinking [28-32] is most often used. Since a third component is not introduced in this approach, the crosslinking of the oligonucleotide remnant can be treated as a type of modification and analyzed by a version of MASCOT. Notwithstanding, there existed a need for a more powerful and useful software package for the analysis of crosslinked products.

**Figure 1:**
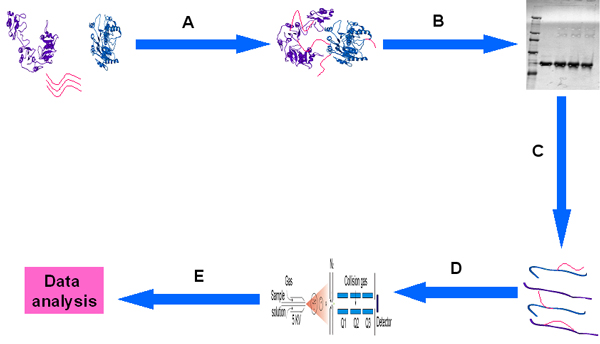
**General strategy of mass spectrometric analysis of protein-protein interactions. **(A) Incubate the crosslinker with the target proteins to acquire crosslinked mixtures. (B) Purify the crosslinked species by SDS-PAGE and (C) extract spots and perform in-gel, protease digestion to yield a peptide pool, which includes crosslinked and uncrosslinked peptides. (D) The peptides are submitted to mass spectrometry to acquire either MS or MS/MS data. (E) Data are subsequently analyzed.

To meet this need, we designed an algorithm, CLPM (Cross-Linked Peptide Map), to map crosslinked peptides derived from a crosslinking reaction of one or two proteins whose sequences are known based on their masses. The general strategy of this approach is shown in Figure 1. The data used as input to this algorithm are the list of mass/charge ratios and the charge of each precursor peak. CLPM has been tested on samples of uncrosslinked proteins, samples of chemically-crosslinked peptides and samples of photochemically-crosslinked protein-DNA and has produced valuable results.

## Implementation

**Figure 2:**
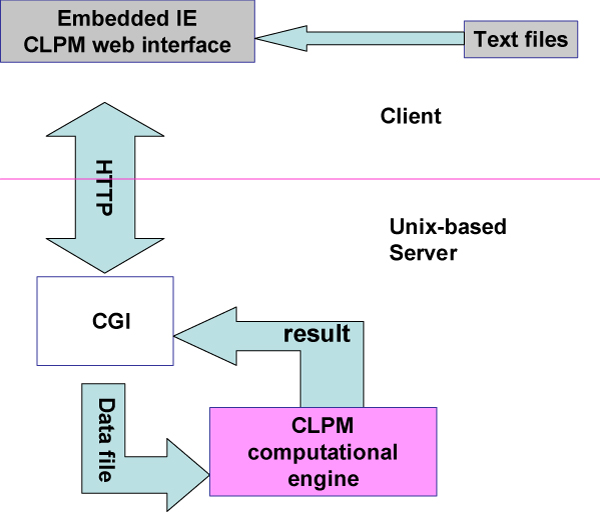
**Structure of CLPM. **Three text files and other information are passed to the server CGI. The CGI reinterprets the data and writes a formatted data file readable by the CLPM computational engine. Results are written to a file and passed back in html format.

**Figure 3:**
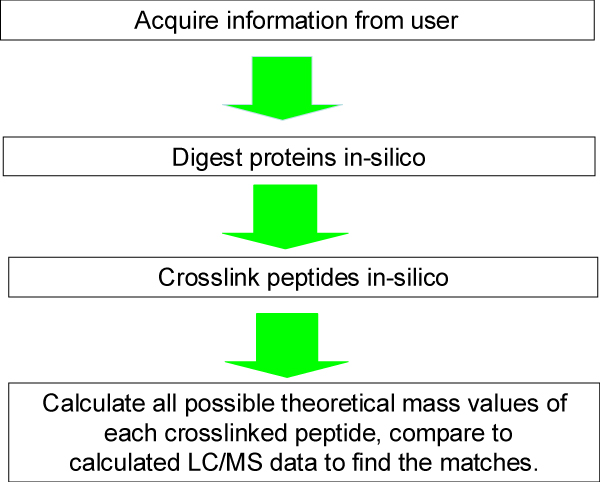
**The workflow of CLPM. **Each step in CLPM is designed to be independent which makes debugging and future extension easier.

CLPM is accessible as an interactive web service though the MidSouth Bioinformatics Center or as a downloadable version upon request. The global structure of this program is shown in Figure 2. Implemented as an object-oriented program in C++, a specific design goal was to allow easy modification and testing. The CLPM computational engine executes according to the workflow shown in Figure 3.

**Figure 4:**
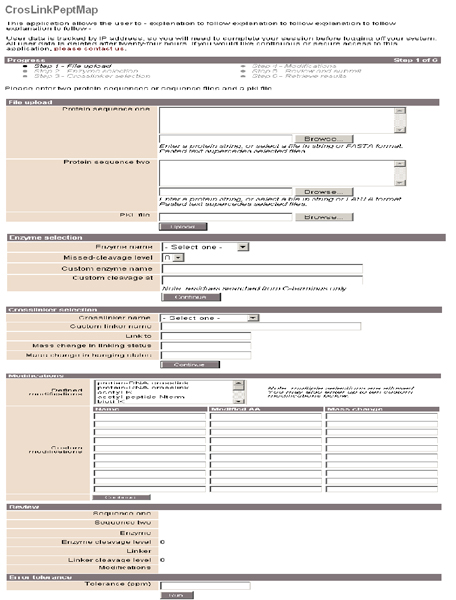
**CLPM web interface. **The sequences and the mass spec data file in pkl format are imported from the user's local system. Users can select enzymes and crosslinkers from a predefined list or define their own custom versions. Users are allowed to select multiple modifications and define as many as ten additional modifications. Missed-cleavage level ranging from zero to five can be selected and the error tolerance is specified in parts per million.

The CLPM provides a simple, but flexible user interface (Figure 4). For the specification of enzymes and crosslinkers, users can either select from a built-in list or define their particular reagents. In the specification of possible chemical modifications, many are available through a built-in list; alternatively the user may define up to ten custom modifications.

Since proteases may not be 100% effective, accommodation needs to be made for missed cleavages. A maximum number of consecutive missed cleavages ranging from 0 to 5 can be specified in CLPM. CLPM assumes a monoisotope mass measurement was performed.

**Figure 5:**
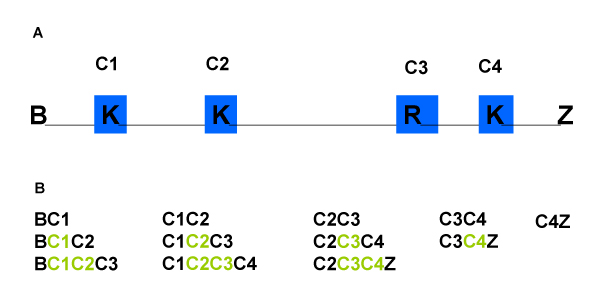
**Mechanism of protein *in silico *digestion. **To digest a protein (A) with four cleavable sites C1 to C4 with a missed-cleavage level of 2, CLPM will generate peptides (B) with zero, one and two consecutively missed cleavage sites (shown in green). B located at the left side of the protein represents the N-terminus of the protein and Z represents the C-terminus.

Because most of the widely-used enzymes digest proteins at the C-terminus of restriction sites, the current version of CLPM only supports these kinds of enzymes. To digest proteins *in silico*, CLPM starts from the N-terminus of each protein sequence and then assumes that the amino acid following each cleavable site is a potential N-terminus of a potential peptide. According to the missed-cleavage level *m*, the C-terminus of each peptide is set to be a cleavable site with *0, 1... m *missed cleavages. All peptides derived from both proteins are stored in string vectors. For a protein with *n *cleavable sites and an *m *missed-cleavage level, there are (*n*+1)*(*m*+1)- [m*(*m*+1)/2] theoretical uncrosslinked peptides generated. The mechanism of protein digestion is shown in Figure 5.

In CLPM, crosslinking is defined as occurring when both arms of a crosslinker are linked to amino acids and this crosslinked entity is treated as a peptide, not a modification. The situation where only one arm of a crosslinker is linked is treated as a modification to the peptide. Crosslinking can occur between peptides (inter-peptide crosslinking) or within a single peptide (intra-peptide crosslinking). Furthermore, inter-peptide crosslinking can be classified as intra-molecular crosslinking or inter-molecular crosslinking; in the former case, the peptides are from different proteins and, in the later case, the peptides are from the same protein. To form intra-peptide crosslinks, CLMP ensures that there are at least two linkable sites within a peptide and the first two linkable sites are used by the crosslinker. To form both types of inter-peptide crosslinks, the first linkable site of each peptide is reserved for the crosslinker. Trypsin, a common enzyme, will not cleave at modified Lys and Arg sites, therefore, peptides with modified C-terminus Lys or Arg are disregarded.

**Figure 6:**
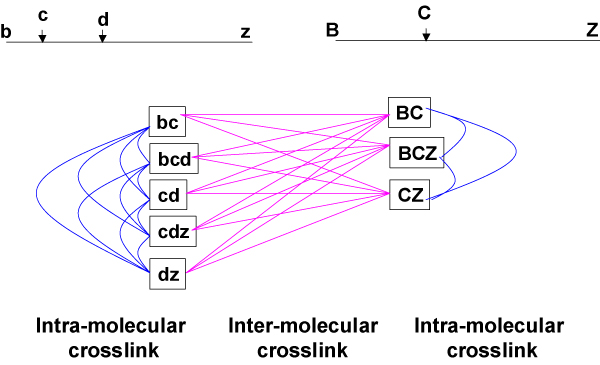
**Mechanism of forming inter-peptides crosslink *in-silico*. **Given two proteins bz and BZ with 2 and 1 cleavable sites respectively and a missed-cleavage level of 1, CLPM will check whether or not there are crosslinking sites in each peptide. For those peptides with crosslinking sites (c and d in bz and C in BZ), CLPM will generate intra-molecule crosslinks (shown in blue) and inter-molecule crosslinks (shown in pink)

The mechanism of generating inter-peptide crosslinked species is illustrated in Figure 6. CLMP generates a complete peptides pool P where

P = {uncrosslinked peptides} U {intra-peptide crosslinked peptides} U {intra-molecular crosslinked peptides} U {inter-molecular crosslinked peptides}

The mass of an unmodified peptide is computed as the mass of its amino acids adjusted as follows (water is gained from hydrolytic digestion):

For uncrosslinked peptides:

mass = sum of masses of all amino acids + mass of water

For intra-peptide crosslinked peptides:

mass = sum of masses of all amino acids + mass of water + mass of crosslinker

For inter-peptide crosslinked peptides:

mass = sum of masses of all amino acids + (2 * mass of water) + mass of crosslinker

CLPM compares the mass of each peptide in P to each observed mass from the mass spectrometric data. When a match is found within the specified maximal mass difference, the search terminates and CLPM moves on to the next peptide in P. If no match is found for an unmodified peptide's mass value, CLPM will then consider its derivatives with modifications. While a peptide may have multiple derivatives, the one encountered with a mass difference which is the smallest among all its derivatives and is within the specified maximum mass difference is considered to be a match.

**Figure 7:**
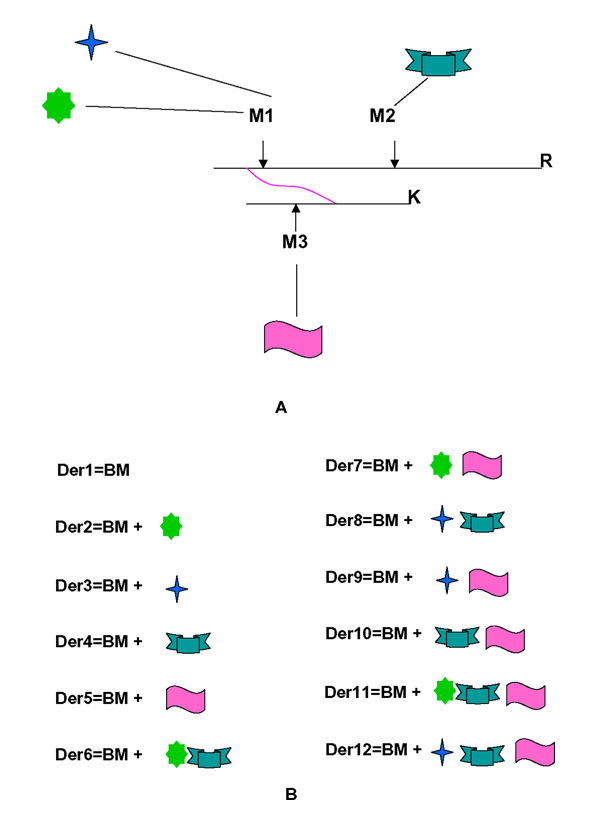
**Mechanism of handling modifications. **(A) For a crosslinked peptide with three modifiable sites M1, M2, and M3, with 2, 1, and 1 possible types of modifications at each site, respectively, CLPM will generate its derivatives. (B) Masses are calculated for each possible combination of derivatives. BM represents the basic mass minus the mass of a peptide without adding any modifications.

For a single peptide with *j *modifiable sites and *x*_*i *_possible modifications at each site *i*, the number of theoretical masses calculated is:



The mechanism to compute derivatives from a peptide is shown in Figure 7.

## Results

### Assessment of CLPM by using an uncrosslinked sample

The MASCOT package was used as a benchmark to validate the basic features of CLPM. Using liquid chromatography (LC) tandem mass spectrometric (MS/MS) data from bovine serum albumin (BSA), CLPM's ability to find crosslinked peptides was compared to that of MASCOT; while a simple test due to MASCOT's limited functionality, this provided a basic initial validation.

Pretryptic uncrosslinked BSA (provided by Dr. Rick Edmondson, NCTR Proteomics Center) 20 uL, 100 Pico mole/uL was loaded through an inline HPLC Micromass Q-TOF Micromass spectrometer (Waters, ) with a gradual increment of organic solvent. Raw data were acquired under positive-ion mode and processed by ProteinLynx 2.0 to generate a data file (.pkl format); this was used as input to MASCOT and CLPM. MASCOT required that carbamidomethylation of Cys be a fixed modification, whereas CLPM allowed it to be treated as a variable modification. In both programs, acetylation of a protein's N-terminus, oxidation of Met, and formation of pyroglutamic acid from N-terminal Glu and Gln were treated as variable modifications. The missed-cleavage level and the error tolerance were set to 2 and 100 ppm, respectively, in both programs. Only monoisotopic values were calculated.

**Figure 8:**
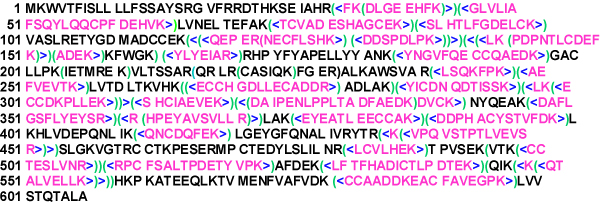
**Comparison of the results of uncrosslinked tryptic BSA by CLPM and MASCOT. **Peptides in <red> were found by MASCOT, while peptides in **() **were found by CLPM. There were 34 peptides found by MASCOT and all were included in the 45 peptides found by CLPM. Settings for the searches were the same except carbamidmethylation of Cys was set as a fixed modification in MASCOT while it was a variable modification in CLPM.

A total of 34 peptides were identified by MASCOT, while 45 peptide matches were found by CLPM. All 34 peptides found by MASCOT were also identified by CLPM; the other 11 peptides were uniquely found by CLPM. Results are shown in Figure 8.

### Assessment of CLPM by crosslinked samples

#### By peptide-peptide crosslinking

A short peptide – AYAGKAGAR – whose N-terminus has been acetylated was chosen to crosslink to the same species with the crosslinker Bis (sulfosuccinimidyl) suberate (BS^3^) so that the result could be confirmed manually. This peptide has been shown to produce good LC/MS/MS data by Hansen, *et al *[26]. The peptide (synthesized by the LSU Core Facility) and BS^3 ^were dissolved in 15 mM PBS buffer in a 2:1 mole-ratio and incubated at room temperature for 60 minutes, then quenched with glycine. Peptides were extracted by reverse-phase ZipTip (Millipore) and eluted into an elution buffer (50% Acetonitrile, 0.1%TFA). LC/MS/MS data were acquired under positive-ion mode. The peptide sequence AYAGKAGAR was used as input to CLPM and peptide acetylation was selected as the only modification.

**Figure 9:**
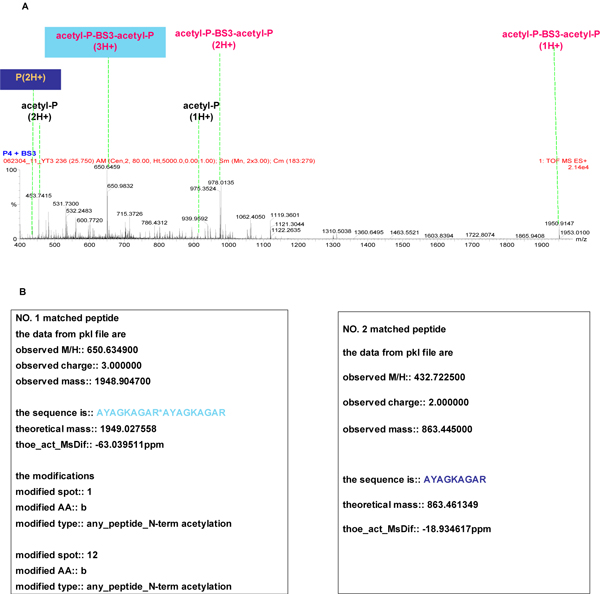
**Result of assessment of CLPM with a crosslinked sample. **(A) BS^3^ was chosen as the crosslinker to crosslink peptide P, whose sequence is acetyl-AYAGKAGAR (in 95% purity). In the MS spectrum, precursor peaks of both crosslinked and uncrosslinked peptides were seen with different charges. (B) CLPM's output shows that an unacetylated peptide was found to match peak 432.7 (2H+) (light blue) and the crosslinked specie of the acetylated peptide was found to match to peak 650.63 (3H+) (dark blue). The output includes the precursor peak, the peptide sequence, the mass of the peptide, the mass-difference between actual and theoretical masses, the modifications (if any), and the possible modified locations.

Both crosslinked and uncrosslinked species were found by CLPM (Figure 9). Surprisingly, the uncrosslinked specie identified was the one without N-terminal acetylation. The LC/MS spectrum is shown in Figure 9A. This "mismatch" came about because (1) unacetylated peptide contaminates acetylated peptide since the latter is only purified to 95% (see the MS spectrum in Figure 9A) and (2) an effect of the "best-match" design of CLPM discussed below.

#### By protein-DNA crosslinking

Hepatitis C Virus Nonstructural Protein 3 (HCV NS3) is a multifunctional enzyme. Its N-terminal 180-amino acid region has protease activity and its C-terminal 465-amino acid region has helicase activity [33,34]. Helicase unwinds DNA or RNA and is one of the core enzymes required for replication, transportation and repair, and recombination. Understanding how the helicase-nucleic acid complex forms is essential for insight into the viral life cycle and for developing treatment for patients with hepatitis C. Tackett *et al *showed that NS3 unwinding activity is sensitive to the structure of the helicase-nucleic acid duplex [35]. A crystal structure of single-stranded DNA (ssDNA) bound NS3 helicase domain (NS3h) has been published. [36] But to our knowledge, no mass spectrometric analysis of the NS3h-ssDNA interaction has been reported. We combined a photochemical crosslinking approach with sequential Q-TOF ESI analysis to acquire the data on the interacting complex. The data were analyzed by CLPM to map the peptide interacting with the ssDNA, and this result was validated by analyzing the spatial relationships within the published crystal structure.

**Figure 10:**
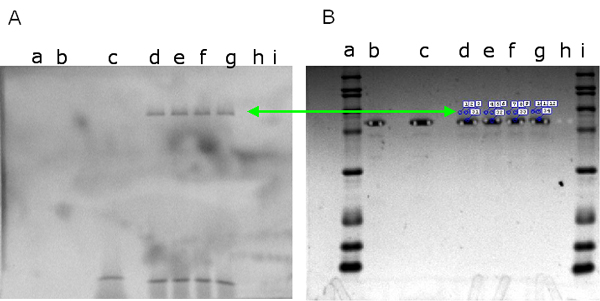
**SDS-PAGE analysis of NS3h-deoxynucleotide photocrosslink. **The crosslink reaction was initiated by exposing the sample to 305 ± 16 nm UV light for three hours. The concentration of DNA was 1 uM and the NS3h concentration was 3 uM. After crosslinking, the sample was separated by SDS-PAGE (12% acrylamide gel). Fluorescent image was taken before (A) and after (B) Commassie staining. Horizontal arrows identify the crosslinked proteins that are 10 kDa heavier than uncrosslinked proteins. Lanes a and i are molecular markers, b is NS3h without adding deoxynucleotide, and c is NS3h and deoxynucleotide without UV light excitation. Lanes d, e, f, and g are NS3h plus deoxynucleotide with three hours activation by 305 nM UV light. Lane h was unused and served as a negative control.

**Figure 11:**
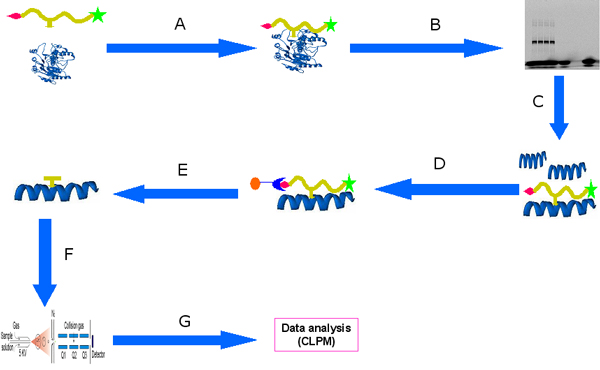
**Procedure to study protein-nucleic acid interactions. **The oligonucleotide is designed to contain a 5'-biotin tag, a 3'-fluorescent tag and a UV sensitive group in the middle. (A) Crosslink initiated by exposing to UV (305+16 nm). (B) After three hours of UV activation, denatured samples were subjected to SDS-PAGE analysis, where crosslinked species were confirmed by fluorescent imaging and all bands were visualized by Commassie staining technique. (C) Interesting spots were picked for protease enzymatic in-gel digestion to yield peptide mixtures. (D) Crosslinked peptides were extracted by magnetic streptavidin beads and uncrosslinked peptides were washed away. (E) The crosslinked peptides were subjected to DNase I degradation to minimize the attaching oligonucleotide moieties. (F) Crosslinked peptides with the remaining nucleic acid attached were extracted by reverse phase ZipTip C18 cartridge and analyzed by Q-tof ESI. (G) Raw data were collected and processed by Protein-Lynx to generate a PKL file, which was used as input to CLPM to identify matches with theoretical peptides.

The study was carried out with the crosslinking of NS3h to a specially-designed DNA 28mer, which contains a 5'-biotin tag, a '3-fluorescent tag and a UV sensitive bromouridine group in the middle. The biotin tag facilitates the extraction of the crosslinked peptide after protease digestion by forming covalent bonds with streptavidin, which is covalently attached to superparamagnetic polystyrene beads. The fluorescent-tag provides an extra method to confirm the crosslinking of the oligonucleotide to the target protein and the bromouridine covalently reacts with oxidizible amino acid sidechains (aromatic or S-containing) when exposed to UV light with wavelengths greater than 300 nm while causing minimal photodamage to other amino acids. Crosslinking was initiated with a ratio of NS3h/DNA 3uM/1 uM. After three hours of UV light excitation, the sample was subjected to fluorescent image and commassie-stained image analysis to confirm the crosslinking (Figure 10), gel-cutting and in-gel trypsin digestion to yield peptide mixtures, extraction to purify crosslinked peptides, DNase digestion to degrade the DNA sequence into short remnants and ESI-MS/MS analysis to acquire the data. The analytic strategy is shown in Figure 11.

**Figure 12:**
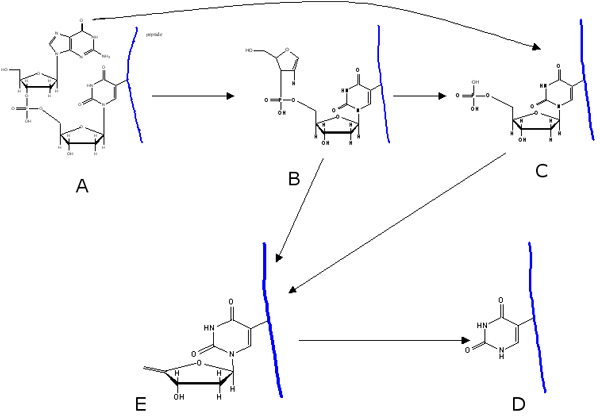
**Possible ion structures for the fragmentation of tryptic peptide crosslinked to the dinucleotide (dGdU) after DNase I digestion as proposed by Golden et al [31]**. The blue line on the right of each diagram represents the peptide moiety in the heteroconjugate.

**Figure 13:**
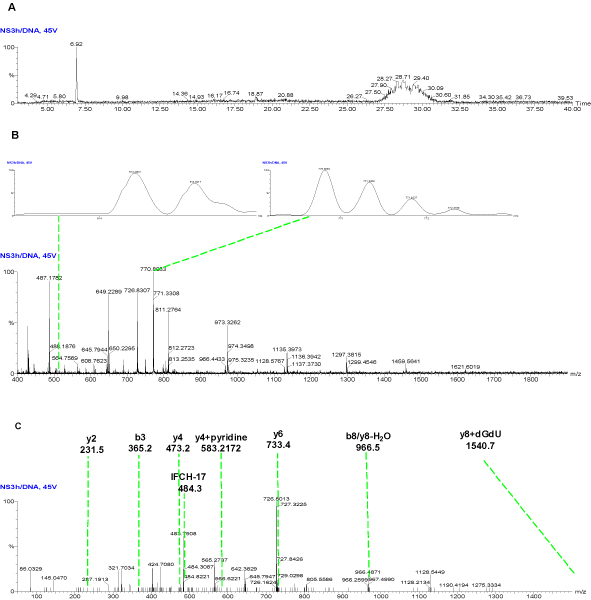
**Chromatography and Mass spectra of peptide HLIFCHSK crosslinking with dGdU. **(A) Chromatograph shows that the peptide eluted at 6.92 minutes. (B) MS spectrum of the crosslinked peptide. CLPM calculated that the doubly-charged precursor ion 770.808 (2H+) has a mass value of 1539.6326Da, which closely matches this crosslinked peptide whose expected mass is 1539.620394Da. The mass difference is 7.93 ppm. The triply-charged ion of this crosslinked peptide can also be found at 514.2 (3H+). In the MS/MS spectrum (C), fragmentation led to some b, y-type and internal fragments of the peptide and of the nucleic acids that were assigned to some of the peaks as shown.

**Figure 14:**
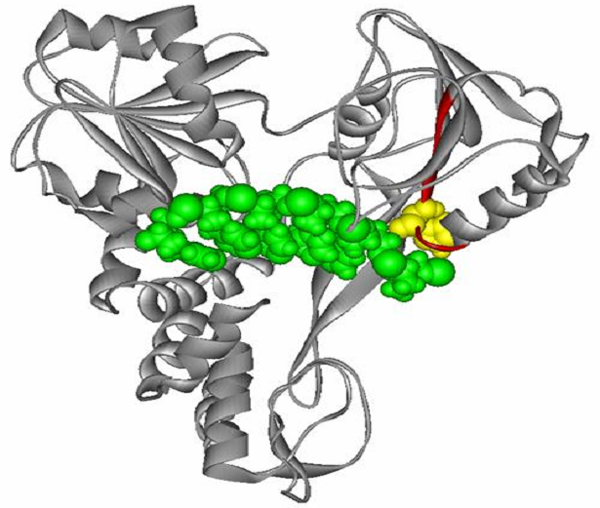
**The locations of H-369 (yellow) and deoxyuridines (green) within the NS3h crystal structure are in close proximity**. H-369 is located within domain 2, and protrudes into the channel where the oligonucleotide binds near the 5' end. A peptide (364–372: HLIFCHSK) (red) was identified by CLPM as the site of photocrosslinking.

During the analysis with CLPM, crosslinking amino acids and the mass changes caused by different types of modifications are defined in CLPM by the ion structures of dinucleotides specified by Golden [31] (Figure 12). The result indicated that the doubly-charged ion with a mass of 770.8163Da matched to a sequence covering the amino acid residues of NS3h protein [36] s from 364 to 372 (HLIFCHSK) with the attachment of a dinucleotide, dGdU, which is considered the "ideal" remnant of deoxyoligonucleotide after being digested by DNase I [30,31]. MS spectrum and MS/MS spectrum of this ion are shown in Figure 13. The cysteine, phenylalanine and two histidines within this peptide can all react with the oligonucleotide. By studying the crystal structure of nucleic acid-bound NS3h, we discerned a very close proximity of H-369 to the deoxyuridine. (Figure 14)

## Discussion

CLPM finds crosslinked peptides derived from crosslinking reactions of one or two known proteins in addition to identifying uncrosslinked peptides. By treating the cleavable crosslinker or oligonucleotide as a type of modification to the peptides, CLPM can also be used to analyze the MS data of crosslinked peptides with cleavable crosslinkers and protein-DNA/protein-RNA heteroconjugates. By doing *in silico *digestion and *in silico *crosslinking, it generates a peptide pool including both crosslinked and uncrosslinked peptides. The mass of each peptide will be compared at least once to the calculated masses of the precursor peaks.

CLPM determines whether or not a precursor peak matches a peptide by calculating the mass-difference between the observed masses and the theoretical masses. If the mass-difference is within a specified error tolerance, then the peptide is considered to match the precursor peak. Starting first with unmodified peptide matches, CLPM tries to avoid false positive hits with the myriad of potential derivatives. If an unmodified peptide is found to be a match with one of the precursor peaks, no further comparisons will be made with any of the potential modified peptides. However, if an unmodified peptide can't be matched to any of the precursor peaks, CLPM will work with different combinations of modifications until a mass-difference within the error tolerance is found, if possible.

In the assessment with an uncrosslinked sample, CLPM successfully mapped all 34 peptides that were found by MASCOT. Further analysis of the 11 peptides uniquely found by CLPM showed that the corresponding precursor peaks were also assigned by MASCOT to other peptides outside the BSA protein and with insignificant scores. The accuracy of MASCOT's results could have been impaired by the low quality of the tandem MS data of those 11 peptides. In other words, the finding of the 11 peptides only by CLPM but not by MASCOT doesn't necessary imply that they are false hits. In the assessment of the sample with a crosslinking reaction, both uncrosslinked and crosslinked peptides were correctly matched to precursor peaks. However, we should note that CLPM does not try to assign each and every observed precursor peak. Rather, CLPM starts with the theoretical peptides derived from the known input sequences. In the case of a peptide with several possible combinations of modifications, only the one with a mass-difference which is within the threshold and is the smallest among all derivates is considered a match. Experience finds this approach greatly reduces the number of false positives. This comes with a disadvantage that only one of several possible peaks which could be assigned to a peptide will be shown as a match.

In the analysis of the NS3h-DNA photochemical crosslinked complex, the possible DNA remnants were treated either as crosslinkers or modifications. In both cases, the same results were derived from CLPM. This result was validated by an X-ray diffraction study, where a very close proximity between H-369 and DNA was shown. A unique challenge was introduced by the DNA moiety: even though some oligonucleotide fragmentation rules under positive-ion mode have been proposed and approved [30-32], there are still many irregularities (e.g., the uncertain number of hydrogen ions maintained by a phosphate group). This greatly increases the complexity of the MS/MS spectrum of peptide-DNA heteroconjugate, which in-turn makes the direct sequence confirmation very difficult. CLPM requires only MS data instead MS/MS data as its input. As long as the mass of DNA moiety was correctly specified, CLPM will map the crosslinked peptide based on its total mass. In the case of uncertain DNA moiety structures, such as in an uncertain degree of DNase degradation, all possible moieties could be selected. CLPM will derive all probable heteroconjugates which can guide further mutagenesis or other corroboration studies.

MS-based methods continue to be the foundation of proteomic analysis [37]. Several other MS-based algorithms such as X-Link [38], NIH-XL [39] and FindLink [40] have been reported to have the abilities to analyze mass spectrometric data of crosslinked reactions. Instead of the best-match approach used in CLPM, X-Link finds all possible crosslinked species solely based on their masses and then identifies the correct species by comparing the expected fragmentation patterns to observed MS/MS data [38]. Moreover, none are freely and publicly available via the Web. To our knowledge, no further detail descriptions of these algorithms have been reported.

As with other MS-based algorithms, the usefulness of CLPM is dependent upon the accuracy of its input: the mass changes due to modifications, and the introduction of crosslinkers, Of course, the inherent accuracy of the mass spectrometry is also a crucial factor. CLPM uses the monoisoptopic values of amino acid residues from [41] and the mass change values due to modifications from [42].

## Conclusion

With the expanding use of mass spectrometry for protein studies comes the need for powerful software to analyze complicated, crosslinked proteins [3]. Identifying peptides by their masses can be an efficient starting point for direct sequence confirmation. The CLPM algorithm provides a powerful tool in identifying these potential interaction sites in combination with chemical crosslinking and mass spectrometry. Through this cost-effective approach, subsequent efforts can quickly focus attention on investigating these specific interaction sites.

## Availability

A free version of CLPM without a graphical interface is available upon request from Yong Tang YxTang2@UALR.edu. This software can be installed on any PC with a minimum requirement of 256 MB RAM. A web-accessible CLPM is available from the MidSouth Bioinformatics Center at: 

## Authors' Contributions

YT designed the algorithm, conducted the experiments validating CLPM, and constructed the web-based version of CLPM. YC assisted with the design of CLPM and helped conduct the bench experiments. CL acquired the Q-TOF ESI mass spectrometry data. KR and SJ supervised the project and provided design oversight. RH and SJ assisted in the construction of the web-based version of CLPM.

## Materials and Methods for HCV NS3 Analysis

The HCV NS3 study was carried out with a specially-designed deoxy-oligonucleotide which contains a photo-sensitive bromouridine group in the middle, a 5'-biotin tag and a 3'-fluorescent tag. The fluorescent tag is 6-carboxyfluorescein (6-FAM) which is a single isomer derivative of fluorescein; it provides an extra method to confirm the crosslinking of the oligonucleotide to the target protein. The 5'-biotin tag facilitates the extraction of the crosslinked peptide after protease digestion by forming covalent bonds with streptavidin which is covalently attached to superparamagnetic polystyrene beads. The bromouridine specifically reacts with oxidizible amino acid sidechains (aromatic or S-containing) when UV light with wavelengths greater than 300 nm is applied with minimum photodamage to certain amino acids. The experiment is diagrammed in Figure 11.

Materials included in this experiment were:

• Photo-reactive deoxy-oligonucleotides

◦ 5'-(Bio)dAdTdG dTdCdG dGdAdT dCdGdC dAdG**/i5Br-dU**/ dCdAdG dTdTdT dTdTdT dTdTdT dT(6-FAM)-3' (stock concentration 144 uM) (synthesized by Dharmacon).

• Recombinant NS3h stored in HEPES buffer.

• Dynabeads M-280 Streptavidin kit (Dynal Biotech).

• DNase I (Ambion).

• HEPES buffer

◦ 25 mM HEPES pH7.5, 50 mM NaCl, 20% Glycerol, 1 mM EDTA, 2 mM BME

Photo-chemical crosslinking was carried out in 25 mM HEPES buffer with a final concentration of oligonucleotides of 1 uM and NS3h of 3 uM (total volume 100 uL).

### Photochemical crosslinking reaction

All reagents were added to a microcentrifuge tube and gently mixed well. The total 100 uL reagent was split into 80 uL for reaction and 20 uL for Control 1 (which contains both the oligonucleotide and the protein but without UV excitation; Control 2 is a 10 uL 3 uM NS3h solution without added oligonucleotide). The 80 uL mixture was transferred to a cuvette and placed into the fluorometer for UV excitation with a wavelength setting of 305 nm and a bandpass setting of 16 nm for three hours. The crosslinking products were added with an equal amount of protein-loading buffer (PSB+BME) and then denatured at 100°C for ten minutes.

### Visualization of crosslinked species and in-gel digestion

SDS-PAGE (sodium dodecyl sulfate polyacrylamide gel electrophoresis) visualization yielded a fluorescent image (before Commassie staining) in which free oligonucleotides and crosslinked species were identified. The same gel was subjected to Commassie stain to visualize the crosslinked and uncrosslinked proteins (Figure 10). Spots of interest were picked by ProPic (Genomic Solutions ) and subjected to in-gel digestion by ProGest (Genomic Solution) (Figure 10). Four hours of trypsin digestion was performed with a typsin/protein ratio of 1:20 (wt/wt) with repeating dehydration, rehydration, reduction and alkylation.

### Enrichment of crosslinked peptides and degradation of oligonucleotides

In order to increase the concentration of peptides, tryptic samples from spots 1 to 6 were combined together. Streptavidins were added to the sample with a ratio of 1 mg streptavidin to 200 pm biotinylated single-stranded oligonucleotide. Uncrosslinked peptides were washed away, but the crosslinked peptides were kept in the tube with the use of a magnet. DNase I was then used to degrade the oligonucleotides with a ratio of 1U DNase I to 1 ug DNA, and the sample was incubated at 37°C in a HEPES buffer for one hour. The supernatant contains the photocrosslinked peptides with the majority of the oligonucleotide digested.

### Mass spectrometry analysis

The crosslinked peptides with the minimized oligonucleotides were extracted with reverse-phase ZipTipC18 (Millipore) and eluted into an elution buffer (50% Acetonitrile, 0.1%TFA) for sequential mass spectrometry analysis. Mass spectrometric data were obtained by Micromass q-TOF Micro spectrometer (Waters) which is connected with a capillary LC system (CapLC, XE, Waters). 20 uL tryptic product was loaded into a ProteoPep C18 column (New Objective). The flow rate was set to 10 uL per minute. Solvent B was gradually increased from 5% during the first minute to 35% at the nineteenth minute, then rapidly increased to 90% at minute 22, then retained at 90% until minute 24. Mass spectra (LC/MS) and tandem mass spectra (LC/MS/MS) were recorded in positive-ion mode. The peptide precursors were automatically selected by the instrument and fragmented in a collision cell using nitrogen as the collision gas and then analyzed by a time-of-flight (TOF) detector.

### Data analyzed by CLPM

Raw data were processed by ProteinLynx to generate a PKL file. The PKL file was used as input to CLPM. The search was done with an error tolerance setting of 100 ppm. Crosslinking amino acids and the mass changes caused by different types of modifications are defined in CLPM by the ion structures of dinucleotides specified by Golden [31] (Figure 12). By comparing this protein-DNA crosslinking with variable modifications including acetylation of a protein's N-terminus, oxidation of Met, and formation of pyroglutamic acid from N-terminal Glu and Gln, CLPM performed exhaustive calculations and comparisons and found the best matches which were defined to have a minimal mass difference from actual masses. Further analysis of the LC/MS/MS spectra of those matched peptides was either done by *de novo *sequencing or by searching against Ms2Assign (free, online software provided by Sandia National laboratories at [43].

## References

[B1] Trester-Zedlitz M, Kamada K, Burley SK, Fenyo D, Chait BT, Muir TW (2003). A modular cross-linking approach for exploring protein interactions. J Am Chem Soc.

[B2] Figeys D, McBroom LD, Moran MF (2001). Mass spectrometry for the study of protein-protein interactions. Methods.

[B3] Sinz A (2003). Chemical cross-linking and mass spectrometry for mapping three-dimensional structures of proteins and protein complexes. J Mass Spectrom.

[B4] Bennett KL, Kussmann M, Bjork P (2000). Chemical cross-linking with thiol-cleavable reagents combined with differential mass spectrometric peptide mapping – a novel approach to assess intermolecular protein contacts. Protein Sci.

[B5] Staub O, Dho S, Henry P (1996). WW domains of Nedd4 bind to the proline-rich PY motifs in the epithelial Na+ channel deleted in Liddle's syndrome. EMBO J.

[B6] Archakov AI, Govorun VM, Dubanov AV (2003). Protein-protein interactions as a target for drugs in proteomics. Proteomics.

[B7] Walsh DM, Klyubin I, Fadeeva J (2002). Naturally secreted oligomers of the Alzheimer amyloid β-protein potently inhibit hippocampal long-term potentiation *in vivo*. Nature.

[B8] Vasilescu J, Guo X, Kast J (2004). Identification of protein-protein interactions using in vivo cross-linking and mass spectrometry. Proteomics.

[B9] Ho Y, Gruhler A, Heilbut A, Bader GD (2002). Systematic identification of protein complexes in Saccharomyces cerevisiae by mass spectrometry. Nature.

[B10] Burgess RR, Thompson NE (2002). Advances in gentle immunoaffinity chromatography. Curr Opin Biotechnol.

[B11] Kellogg DR, Moazed D (2002). Protein-and immunoaffinity purification of multiprotein complexes. Methods Enzymol.

[B12] Zhao Y, Muir TW, Kent SBH, Tischer E, Scardina JM, Chait BT (1996). Mapping protein-protein interactions by affinity-directed mass spectrometry. Proc Natl Acad Sci.

[B13] Farmer TB, Caprioli RM (1998). Determination of Protein-Protein Interactions by Matrix-assisted Laser Desorption/Ionization Mass Spectrometry. J Mass Spectrom.

[B14] Swietnicki W, O'Brien S, Holman K (2004). Novel protein-protein interactions of the Yersinia pestis type III secretion system elucidated with a matrix analysis by surface plasmon resonance and mass spectrometry. J Biol Chem.

[B15] Kriwacki RW, Siuzdak G (2000). Probing protein-protein interactions with mass spectrometry. Methods Mol Biol.

[B16] Mann M, Hendrickson RC, Pandey A (2001). Analysis of proteins and proteomes by mass spectrometry. Annu Rev Biochem.

[B17] Ayed A, Krutchinsky AN, Ens W, Standing KG, Duckworth HW (1998). Quantitative evaluation of protein-protein and ligand-protein equilibria of a large allosteric enzyme by electrospray ionization time-of-flight mass spectrometry. Rapid Commun Mass Spectrom.

[B18] Yang T, Horejsh DR, Mahan KJ, Zaluzec EJ, Watson TJ, Gage DA (1996). Mapping cross-linking sites in modified proteins with mass spectrometry: an application to cross-linked hemoglobins. Anal Biochem.

[B19] Back JW, Hartog AF, Dekker HL, Muijsers AO, de Koning LJ, de Jong L (2001). A new crosslinker for mass spectrometric analysis of the quaternary structure of protein complexes. J Am Soc Mass Spectrom.

[B20] Cai K, Itoh Y, Khorana HG (2001). Mapping of contact sites in complex formation between transducin and light-activated rhodopsin by covalent crosslinking: use of a photoactivatable reagent. Proc Natl Acad Sci U S A.

[B21] Itoh Y, Cai K, Khorana HG (2001). Mapping of contact sites in complex formation between light-activated rhodopsin and transducin by covalent crosslinking: use of a chemically preactivated reagent. Proc Natl Acad Sci U S A.

[B22] Young MM, Tang N, Hempel JC (2000). High throughput protein fold identification by using experimental constraints derived from intramolecular cross-links and mass spectrometry. Proc Natl Acad Sci U S A.

[B23] Novak P, Young MM, Schoeniger JS, Kruppa GH (2003). A top-down approach to protein structure studies using chemical cross-linking and Fourier transform mass spectrometry. Eur J Mass Spectrom (Chichester, Eng).

[B24] Schilling B, Row RH, Gibson BW, Guo X, Young MM (2003). MS2Assign, automated assignment and nomenclature of tandem mass spectra of chemically crosslinked peptides. J Am Soc Mass Spectrom.

[B25] Perkins DN, Pappin DJ, Creasy DM, Cottrell JS (1999). Probability-based protein identification by searching sequence databases using mass spectrometry data. Electrophoresis.

[B26] Hansen BT, Jones JA, Mason DE, Liebler DC (2001). SALSA: a pattern recognition algorithm to detect electrophile-adducted peptides by automated evaluation of CID spectra in LC-MS-MS analyses. Anal Chem.

[B27] ASAP. http://roswell.ca.sandia.gov/~mmyoung/asap.html.

[B28] Gott JM, Willis MC, Koch TH, Uhlenbeck OC (1991). A specific, UV-induced RNA-protein cross-link using 5-bromouridine-substituted RNA. Biochemistry.

[B29] Wong DL, Pavlovich JG, Reich NO (1998). Electrospray ionization mass spectrometric characterization of photocrosslinked DNA-EcoRI DNA methyltransferase complexes. Nucleic Acids Res.

[B30] Steen H, Jensen ON (2002). Analysis of protein-nucleic acid interactions by photochemical cross-linking and mass spectrometry. Mass Spectrom Rev.

[B31] Golden MC, Resing KA, Collins BD, Willis MC, Koch TH (1999). Mass spectral characterization of a protein-nucleic acid photocrosslink. Protein Sci.

[B32] Steen H, Petersen J, Mann M, Jensen ON (2001). Mass spectrometric analysis of a UV-cross-linked protein-DNA complex: tryptophans 54 and 88 of E. coli SSB cross-link to DNA. Protein Sci.

[B33] Borowski P, Kuhl R, Laufs R, Schulze zur Wiesch J, Heiland M (1999). Identification and characterization of a histone binding site of the non-structural protein 3 of hepatitis C virus. J Clin Virol.

[B34] Kwong AD, Kim JL, Lin C (2000). Structure and function of hepatitis C virus NS3 helicase. Curr Top Microbiol Immunol.

[B35] Tackett AJ, Wei L, Cameron CE, Raney KD (2001). Unwinding of nucleic acids by HCV NS3 helicase is sensitive to the structure of the duplex. Nucleic Acids Res.

[B36] Kim JL, Morgenstern KA, Griffith JP (1998). Hepatitis C virus NS3 RNA helicase domain with a bound oligonucleotide: the crystal structure provides insights into the mode of unwinding. Structure.

[B37] Henzel WJ, Watanabe C, Stults JT (2003). Protein identification: the origins of peptide mass fingerprinting. J Am Soc Mass Spectrom.

[B38] Taverner T, Hall NE, O'Hair RA, Simpson RJ (2002). Characterization of an antagonist interleukin-6 dimer by stable isotope labeling, cross-linking, and mass spectrometry. J Biol Chem.

[B39] Sinz A, Wang K (2001). Mapping protein interfaces with a fluorogenic cross-linker and mass spectrometry: application to nebulin-calmodulin complexes. Biochemistry.

[B40] Back JW, Sanz MA, De Jong L (2002). A structure for the yeast prohibitin complex: Structure prediction and evidence from chemical crosslinking and mass spectrometry. Protein Sci.

[B41] IonSource. http://www.ionsource.com.

[B42] UNIMOD protein modifications for mass spectrometry. http://www.unimod.org/.

[B43] Ms2Assign. http://roswell.ca.sandia.gov/~mmyoung/ms2assign.html.

